# Assessment of Myocardial Scar; Comparison Between ^18^F-FDG PET, CMR and ^99^Tc-Sestamibi

**DOI:** 10.4137/cmc.s730

**Published:** 2009-06-08

**Authors:** Andrew Crean, Sadia N. Khan, L. Ceri Davies, Richard Coulden, David P. Dutka

**Affiliations:** 1 Cardiovascular Medicine, Department of Medicine, University of Cambridge and; 2 Department of Radiology, Papworth Hospital, Papworth Everard, Cambridge, England.

**Keywords:** heart failure, hibernation, PET, MIBI, CMR

## Abstract

**Objective:**

Patients with heart failure and ischaemic heart disease may obtain benefit from revascularisation if viable dysfunctional myocardium is present. Such patients have an increased operative risk, so it is important to ensure that viability is correctly identified. In this study, we have compared the utility of 3 imaging modalities to detect myocardial scar.

**Design:**

Prospective, descriptive study.

**Setting:**

Tertiary cardiac centre.

**Patients:**

35 patients (29 male, average age 70 years) with coronary artery disease and symptoms of heart failure (>NYHA class II).

**Intervention:**

Assessment of myocardial scar by ^99^Tc-Sestamibi (MIBI), ^18^F-flurodeoxyglucose (FDG) and cardiac magnetic resonance (CMR).

**Outcome Measure:**

The presence or absence of scar using a 20-segment model.

**Results:**

More segments were identified as nonviable scar using MIBI than with FDG or CMR. FDG identified the least number of scar segments per patient (7.4 +/− 4.8 with MIBI vs. 4.9 +/− 4.2 with FDG vs. 5.8 +/− 5.0 with CMR, p = 0.0001 by ANOVA). The strongest agreement between modalities was in the anterior wall with the weakest agreement in the inferior wall. Overall, the agreement between modalities was moderate to good.

**Conclusion:**

There is considerable variation amongst these 3 techniques in identifying scarred myocardium in patients with coronary disease and heart failure. MIBI and CMR identify more scar than FDG. We recommend that MIBI is not used as the sole imaging modality in patients undergoing assessment of myocardial viability.

## Introduction

Coronary artery disease is the most common cause of heart failure in the Western world.^1^–^3^ It is increasingly recognised that myocardium, which was once thought to be irreversibly damaged, may recover function and hence improve prognosis and symptoms in selected patients who undergo revascularisation in addition to optimal medical management.^4^ Clinically, this relies on myocardial viability assessment using imaging techniques. Decisions regarding high risk revascularisation also need to be made, sometimes urgently, when patients presenting in NYHA class 3/4 heart failure have significant left ventricular dysfunction and coronary artery disease yet do not have a history of limiting angina.

Patients with impaired left ventricular function represent a higher operative risk group than patients with normal LV function. Recent reports indicate that the perioperative mortality after CABG in patients with advanced LV dysfunction varies between 2.5%–8%. This is compared to standard CABG mortality of less than 2%. In addition, LV dysfunction is a variable on standard scoring systems for assessing likely mortality such as the Euroscore/Parsonnett systems.^5^–^8^ There are suggestions from non randomized observational data that patients with dysfunctional but viable myocardium are more likely to survive if they undergo revascularisation as opposed to medical therapy. Various imaging modalities have been proposed to detect viability, including nuclear techniques and cardiac magnetic resonance imaging (CMR).^9^ Each technique has advantages, some of which are determined by patient characteristics. The identification of viable dysfunctional myocardium falsely imaged as scar is a concern as this can adversely influence treatment decisions. This is a particular problem in subjects with significant left ventricular dysfunction, as this group potentially have the most to gain from revascularisation^10^ and in whom perfusion and wall motion defects are most likely to be present. In this study, we report our clinical experience in a group of patients referred for viability assessment who underwent testing with three commonly used imaging modalities, ^99^Tc-Sestamibi (MIBI), ^18^F-flurodeoxyglucose (FDG) and CMR, with a particular emphasis on the identification of scar by each imaging modality.

## Methods

Subjects were referred for viability assessment if the assessing clinician felt that the patients might benefit from intervention for coronary artery disease in the context of clinical heart failure. Medical management was optimised and all were clinically stable prior with at least NYHA class II symptomatic heart failure. Forty six patients were studied over a 24 month period. MR scans were not undertaken in 4 subjects with implanted devices (pacemakers or defibrillators) and 3 were too large for the modified gamma camera. The images obtained from four of the FDG scans were not of diagnostic standard and were classed as technical failures such that complete data from all three imaging techniques was available for 35 patients. The baseline characteristics are shown in Table 1. Mean ejection fraction by MRI was 24% (median 23%) with a range of 10%–56%. Mean end diastolic volume was 200 ml with a range of 105–455 ml.

Assessments were undertaken on separate days according to the standard clinical protocol described below. None of the subjects reported a clinical event during the period of testing. The studies were reviewed by at least two cardiologists/ radiologists blinded to clinical data. A 20-segment model was used with 2 apical segments, and 3 radial layers of 6 segments at apical, mid and basal levels using short axis slices. This model was chosen as the software package for SPECT studies in our institution generates a 20 segment model and the AHA consensus statement supports a 20 segment model for this technique.^11^ Images were compared to determine agreement between areas of scar identified by each technique.

### Sestamibi scanning

A two-day protocol was used with stress and rest scans at least 24 hours apart. Adenosine was used in the majority of cases as the stress agent at a dose of 140 mcg/kg/min for a period of 6 minutes, with tracer being injected at 3 minutes. Dobutamine was used as an alternative stress agent in subjects with significant airways disease. Sublingual nitrates were administered in all cases prior to the isotope injection during the rest study. ^99m^Tc MIBI (400 mBq) was administered for both the rest and the stress studies and images acquired with gated SPECT on a dual headed gamma camera (GE Millenium VG) at least 90 minutes post administration of radiopharmaceutical. Acquisition was performed over a 360 degree arc using a standard parallel hole collimator. Subsequent data reconstruction and analysis was performed with QPS/QGS software (Cedars-Sinai, CA) for production of bulls-eye plots/gated EF assessment respectively, on a dedicated Entegra workstation (GE Healthcare, UK). Ejection fraction was obtained from the gated rest images. Viability was defined as uptake >60% of maximal pixel intensity on the resting image as described in the CHRISTMAS trial.^12^

### ^18^F-FDG scanning

Subjects were fasted for at least 6 hours. A hyperinsulinaemic euglycaemic clamp was performed to standardise metabolic conditions and to maximise myocardial ^18^F-fluorodeoxyglucose (FDG) uptake. The average glucose infusion rate during the equilibrium phase of the clamp was 4.2+/−1.7 mg/kg/min. At least 80 minutes after commencement of insulin (during clamp steady state), FDG (185 MBq) was injected and scanning performed 30 minutes after injection of isotope. Images were acquired on the same gamma camera as the MIBI studies using high energy collimators to facilitate coincidence mode, and corrected for attenuation using X-rays. Images were analysed using an Entegra workstation and Cedars-Sinai software (GE medical systems). A segment was considered viable if uptake was >50% of the maximum pixel intensity.^13^

## CMR

Patients were imaged using a 1.5T GE Signa CV/i scanner (GE Healthcare, UK) with a four channel cardiac phased array coil. Cine images were acquired by steady state free precession imaging in the short axis, 2 chamber and 4 chamber cardiac planes (slice thickness 10 mm, interslice gap 0–3 mm depending upon length of ventricle). Technical parameters: TR 3.5, TE 1.3, flip angle 45 degrees, 0.75 NEX, matrix 224 × 128, FOV 36 × 36 cm, 75% phase field of view.

Viability imaging (“delayed enhancement”) was performed 10–30 minutes following a total bolus of 0.2 mmol/kg of gadolinium chelate (Omniscan, Amersham NJ) using an inversion recovery-prepared gradient echo pulse sequence. The optimal inversion time was individually adjusted for each patient. Images were acquired in matching locations to the cine images. Technical parameters: TR 7.2, TE 3.2, flip angle 15 degrees, matrix 256 × 192, trigger delay 500 msec, 75% phase field of view, inversion time individually matched to patient (range 180–220 msec).

Polar viability maps were constructed based using the same 20 segment model as above, and viability defined as wall thickness >6 mm without delayed gadolinium enhancement.^14^ In areas of borderline wall thickness the segment was judged to be scar if the transmural extent of gadolinium enhancement was >25%.^15^

Volumetric measurements from the MR data set were made by importing the short axis steady state free precession cine images into a dedicated off line workstation with appropriate software (Advantage Windows 4.2, GE Medical Systems, Milwaukee; Mass Analysis Plus 5.2, MEDIS Medical Imaging Systems, Nuenen, Netherlands).

### Statistical methods

Statistical calculations were performed using Statview 4.5 (Abacus Concepts, Berkeley, CA, USA). Numerical values are presented as mean +/− standard deviation. Comparisons between group means were carried out by analysis of variance (ANOVA). The agreement between the 3 imaging modalities to detect cardiac scar on a segment by segment basis was assessed using κ statistics. K values of less than 0.4, of 0.4 to 0.75 and greater than 0.75 were considered to represent poor, moderate to good and excellent agreement respectively on the basis of the Fleiss classification.^16^ A p value < 0.05 was considered statistically significant.

## Results

The total number of scarred segments per patient for each imaging modality is shown in Table 2.

A significantly higher number of segments were identified as scar by MIBI than by either FDG or CMR. The over-estimation of scar by MIBI was independent of site within the left ventricle as shown in Table 3. For all modalities, apical segments were included in the anterior wall.

**Figure 1.**
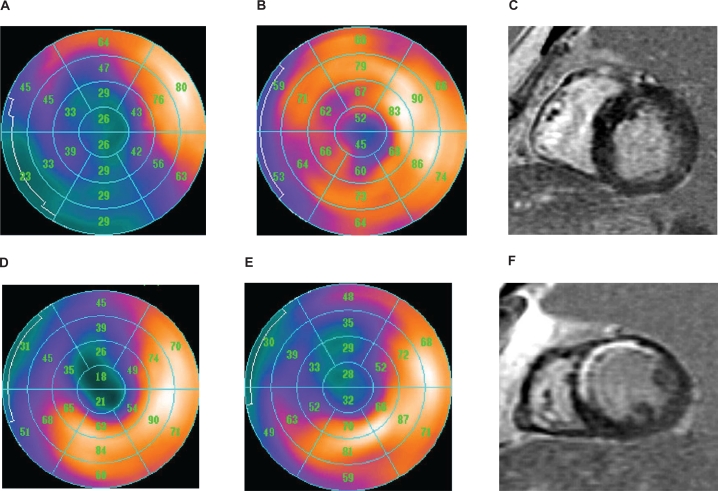
Upper panel: a discordant study with inferior scar identified with MIBI, but viable myocardium with FDG and CMR (**A**–**C**). Lower panel: example of a concordant study with anterior scar identified with all three imaging modalities (**D**–**F**).

The best agreement between modalities was seen in the anterior wall with virtually no difference in the number of scar segments seen by MIBI, FDG and CMR. The worst agreement occurred in the inferior wall with an 87% difference between MIBI and FDG. An example of this discrepancy is shown in Figure 1(a), where MIBI imaging shows a large scar in the infero-lateral wall of the left ventricle which is absent on both FDG and CMR. Scar segments were seen most frequently in the inferior wall with MIBI scanning but in the anterior wall by both FDG and CMR. Concordant images with anteroseptal scar are shown in Figure 1(b).

The patients were divided into tertiles on the basis of resting ejection fraction determined from the gated MIBI scan. As might be expected, those patients with more marked left ventricular dysfunction demonstrated significantly more scar than those with more preserved ventricular function (Table 4). Again the discrepancy between amount of scar identified by the three modalities was maintained, except for patients with the highest ejection fraction, where CMR demonstrated least scar.

## Discussion

### 

There is an increasing evidence base to support revascularisation in the context of viable ischaemic myocardium.^17^ Clinically this relies on the use of imaging to accurately delineate scarred (infarcted) myocardium from viable, non-functioning myocardium. Each of the imaging modalities used by our department is discussed in the recent report from the European Society of Cardiology.^9^ Our results would suggest that there is considerable variation amongst these commonly used techniques. For clinical use in perfusion assessment, technetium based tracers are often preferred to thallium as these tracers result in lower radiation exposure to the patient and a reduction in soft tissue attenuation.^18^ However, in common with others we have found that MIBI overestimates areas of myocardial scar tissue.^19^–^24^ Our patient cohort had moderate to severe left ventricular dysfunction but these differences were seen across all tertiles of left ventricular function suggesting that increasing left ventricular impairment alone cannot account for the differences observed. Technetium MIBI appeared to overestimate scar most frequently in the inferior LV segments, presumably as a result of diaphragmatic attenuation. Recent data has shown that attenuation correction can increase agreement between technetium based tracers and FDG-SPECT,^25^ particularly when assessing viability in the infero-posterior wall. However, in our group of patients, differences were also found in the imaging of the lateral wall suggesting that this cannot completely explain these results. Our findings would bring into question the use of technetium MIBI to select subjects as having evidence of hibernation (for example as in the CHRISTMAS trial)^12^ as our data suggests that many segments, which would seem to be scarred, are in fact viable by other imaging techniques. The over-reporting of scar by MIBI is unlikely to be explained solely by changes in perfusion at rest,^26^ as it is known that hibernating myocardium has preserved blood flow at rest.^27^ Reduced uptake/enhanced washout of technetium based tracers has been reported^28^,^29^ and it has been postulated that repetitive ischaemia might contribute to this.^29^ This mechanism is also thought to be important in chronic left ventricular dysfunction secondary to coronary artery disease^30^ and we believe that this may account for the some of the differences observed here. Based on these results, it may be prudent to consider alternatives to technetium MIBI in the assessment of myocardial viability.

There is a relatively close agreement between FDG and CMR although variation exists between these two modalities that may reflect the limited spatial resolution and/or partial volume effects associated with FDG imaging using a modified gamma camera that would tend to limit delineation of scar.^31^ Previous investigators have shown that visual analysis of MR has good correlation with quantitative analysis^32^ so we do not believe that this is solely a reflection of visual analysis although clearly image alignment with different imaging modalities remains a concern. Although there is numerical discrepancy in approximately half of all studies, clinical review of these patients suggests that this difference would have altered management in less than 5 cases. The discrepancy is unlikely to be a reflection of the FDG-SPECT technique *per se*. Only 2 out of 35 patients were diabetic and excellent suppression of myocardial FFA utilisation was achieved in all patients with the insulin clamp method. Whilst preserved myocardial metabolic activity using FDG during hyperinsulinaemia is often regarded as the gold standard to assess viability, our findings suggest that there is no clinically significant difference between FDG imaging and CMR for viability assessment. We chose to define non viability at CMR primarily by end diastolic wall thickness and used presence of delayed enhancement as a secondary criterion. This is because there remains some controversy about the exact degree to which trans-mural extent of scar identified by gadolinium correlates with non recoverability of function. It can also be difficult to accurately delineate quartiles of transmurality in regions of severely thinned myocardium where an overall measurement of end diastolic wall thickness is potentially more reliable and reproducible. Although delayed enhancement at CMR may reflect uptake into an expanded interstitial space in addition to delayed washout from scar following acute myocardial infarction, this consideration was not relevant to our sample since no patients were studied within 6 months of an acute coronary event and the majority were suffering from chronic ischaemic cardiomyopathy.

One aspect of this work requiring consideration is the homogeneity of our patient population. A small number of patients had minor atheroma or only single vessel disease raising the question of whether they were incorrectly classified as ischaemic rather than dilated cardiomyopathy. However it is well accepted that infarction frequently occurs on low grade stenoses and a subendocardial pattern of infarction characteristic of coronary disease was seen by delayed enhancement CMR in all but one of these patients. The ability of CMR to distinguish between heart failure secondary to coronary disease from that due to other forms of cardiomyopathy has been well described previously.^33^ Only one subject displayed midwall septal enhancement without subendocardial enhancement elsewhere and we believe that this was an isolated case of idiopathic rather than ischaemic cardiomyopathy.

#### Study limitations

In this study we used end diastolic wall thickness (EDWT) as our major determinant of viability by MRI. It is generally accepted that transmural extent of late gadolinium enhancement (LGE) is the gold standard for determining myocardial viability. However, the LGE technique is more challenging in the severely breathless heart failure patient due to altered blood pool kinetics and limited achievable spatial resolution (due to impaired patient breath hold ability) in areas of myocardial thinning. A navigated free-breathing approach to LGE image acquisition was discounted because of the length of time necessary to acquire a full volume data set in these enlarged left ventricles. Nonetheless EDWT is a reasonable surrogate that has been used many times in earlier literature and remains a valid measure.

The limitations of this study also include the relatively small number of patients assessed and the lack of attenuation correction as discussed above. This is likely to increase the agreement between MIBI and the other imaging modalities but should not influence the agreement between FDG-SPECT and CMR. This can only be clarified by defining the extent of functional recovery following revascularisation, which we believe is the most significant limitation of this study.

In summary we present data obtained in a clinical service using three common imaging techniques in a group of patients. We suggest that technetium MIBI should not be used to define myocardial viability where more accurate methods exist. Local resource availability and patient characteristics may well dictate the use of FDG-PET or CMR and our data suggest that there is little practical difference between these two modalities.

## Figures and Tables

**Table 1.** Patients’ baseline characteristics.

Age (mean ± SD)	70 ± 9
Sex	29 males 6 females
Diabetes	2
Triple vessel disease	24
Two vessel disease	3
Single vessel disease	4
Minor atheroma only	4
Ejection fraction by MRI (mean ± SD)	24 ± 12%
Ejection fraction by MIBI (mean ± SD)	33 ± 11%
Patients with documented prior MI	16
Within 6–12 months	5
Greater than 12 months	11
Body mass index (mean ± SD)	28 ± 3.8 kg/m^2^
Fasting blood glucose (mean ± SD)	6.1 ± 1.78 mmol/l
Resting systolic blood pressure (mean ± SD)	141.8 ± 20.3 mmHg
Resting diastolic blood pressure (mean ± SD)	85.8 ± 12.0 mmHg

**Table 2.** Comparison of scar burden between modalities.

	**Number of segments classified as scar**		
	**MIBI**	**MRI**	**FDG**
	7.4 ± 4.8	5.8 ± 5	4.9 ± 4.2
	**MIBI vs. MRI**	**MIBI vs. FDG**	**MRI vs. FDG**
Difference in mean number of segments (95% CIs)	1.57 (0.61–2.53)	2.46 (1.27–3.64)	−0.89 (−2.06 – 0.27)
P statistic	p = 0.002	p = 0.0002	p = 0.13
Weighted kappa	κ = 0.38	κ = 0.48	κ = 0.77

**Table 3.** Average number of scar segments per patient according to the cardiac location.

	**MIBI**	**CMR**	**FDG**	**P**
Anterior	2.1 +/− 1.9	2.0 +/− 1.9	2.0 +/− 1.7	0.6
Septum	0.9 +/− 1.2	0.8 +/− 1.1	0.9 +/− 1.2	0.5
Lateral	1.6 +/− 1.6	1.1 +/− 1.4	0.6 +/− 1.1	<0.0001
Inferior	2.8 +/− 2.1	1.9 +/− 1.9	1.5 +/− 1.8	<0.0001

**Table 4.** Average number of scar segments per patient according to ejection fraction.

**EF**	**<28%**	**>28% <41%**	**>41%**
PET	7.6 +/− 3.8	4.6 +/− 3.9	2.5 +/− 3.5
MIBI	73.2 +/− 3.0	7.5 +/− 4.7	3.2 +/− 3.0
CMR	6.3 +/− 4.61	6.3 +/− 4.6	1.2 +/− 2.3
